# A Multivariable Index for Grading Exercise Gas Exchange Severity in Patients with Pulmonary Arterial Hypertension and Heart Failure

**DOI:** 10.1155/2012/962598

**Published:** 2012-12-31

**Authors:** Chul-Ho Kim, Steve Anderson, Dean MacCarter, Bruce Johnson

**Affiliations:** Division of Cardiovascular Diseases, Mayo Clinic, Rochester, MN 55905, USA

## Abstract

Patients with pulmonary arterial hypertension (PAH) and heart failure (HF) display many abnormalities in respiratory gas exchange. These abnormalities are accentuated with exercise and track with disease severity. However, use of gas exchange measures in day-to-day clinical practice is limited by several issues, including the large number of variables available and difficulty in data interpretation. Moreover, maximal exercise testing has limitations in clinical populations due to their complexity, patient anxiety and variability in protocols and cost. Therefore, a multivariable gas exchange index (MVI) that integrates key gas exchange variables obtained during submaximal exercise into a severity score that ranges from normal to severe-very-severe is proposed. To demonstrate the usefulness of this index, we applied this to 2 groups (PAH, *n* = 42 and HF, *n* = 47) as well as to age matched healthy controls (*n* = 25). We demonstrate that this score tracks WHO classification and right ventricular systolic pressure in PAH (*r* = 0.53 and 0.73, *P* ≤ 0.01) and NYHA and cardiac index in HF (*r* = 0.49 and 0.74, *P* ≤ 0.01). This index demonstrates a stronger relationship than any single gas exchange variable alone. In conclusion, MVI obtained from light, submaximal exercise gas exchange is a useful approach to simplify data interpretation in PAH and HF populations.

## 1. Introduction

The lungs are linked hemodynamically in series with the heart, share a common surface area, are exposed to similar intrathoracic pressure changes during breathing, compete for intrathoracic space, and receive nearly 100% of the cardiac output. Receptors in the heart influence breathing patterns, while neural pathways in the lungs in turn may influence cardiac function (e.g., heart rate). Small increases in metabolic demand (e.g., exercise) enhance these cardiopulmonary interactions. Thus it is no surprise that diseases that primarily influence the lungs or the heart significantly impact the other organ system [1, 2]. This can be especially observed in patients with pulmonary arterial hypertension (PAH) where right heart failure evolves and in patients with left heart failure (HF) where significant changes occur in lung mechanics, ventilatory control, and ultimately in respiratory gas exchange. In both these patient groups gas exchange abnormalities are often present at rest, but are accentuated with the challenges of exercise. Thus, noninvasive measures of cardiopulmonary gas exchange obtained during exercise have become a relatively common means to assess disease severity, prognosis, and response to therapy. However, despite the large availability of data confirming the utility of exercise gas exchange measures during exercise in these patients groups and the quickly improving and simplified approaches to testing, noninvasive respiratory gas exchange remains relatively poorly understood and underutilized in day to day clinical practice [3].

There have been a number of impediments to more extensive utilization of exercise respiratory gas exchange. This includes issues such as the large number of variables that are produced from typical commercially based systems, the somewhat broad range of normal values (influenced by age, gender, fitness, obesity, anxiety, body size, etc.), comorbidities that may influence the data, the complexities and expense that have been associated with comprehensive clinically based cardiopulmonary exercise testing, and difficulties and anxieties associated with maximal testing of often brittle patient populations [4]. 

However, noninvasive commercially available gas exchange systems have been developed that are simpler, self-calibrating, with a lighter, less complicated patient interface [5]. In addition, it is becoming clear that gas exchange data other than peak oxygen consumption (*V*O_2max⁡_ or *V*O_2peak_) that can be obtained from light or submaximal exercise (e.g., *V*_*E*_/*V*CO_2_ slope, OUES, and PetCO_2_) as a slope or change from rest may be as good or in some cases more prognostic, reproducible, and sensitive than those obtained from maximal exercise testing and provoke less patient anxiety at reduced cost [6]. We have previously demonstrated that blending simpler devices with minimized, and submaximal protocols is well liked by patients, with the gas exchange data adequately separating both PAH and HF patients from healthy populations and according to disease severity [7–9].

To further simplify cardiopulmonary gas exchange for clinical use in the PAH and HF populations, we are further proposing a multivariable index (MVI) that takes into account the key gas exchange variables obtained during exercise that have been shown to be associated with these disease entities. The value of a multivariable index, or score, has been previously suggested and should have the following characteristics [10]: (1) utilizes variables that have been well documented in the literature for their normative ranges and prognostic value, (2) utilizes variables that have been associated with other clinical identifiers (e.g., disease classifications or common clinical metrics such as right heart pressures or cardiac index), (3) utilizes a model that can easily be adjusted as literature evolves, and (4) provides a simple conceptual framework for scoring that is similar to clinically intuitive scoring methods (e.g., WHO or NYHA classification), but provides a continuous variable which is more sensitive to changes in disease pathophysiology or to therapy than typical, more subjective scoring systems. This approach to creating a novel noninvasive gas exchange severity score from submaximal data for both PAH and HF is described and tested in these patient groups. We previously reported a gas exchange scoring system specific for PAH; however, we suggest this current more comprehensive and systematic approach provides a clearer framework for tracking PAH patients, appears to track disease status in the HF population, and provides a modifier for exercise induced PH [11–13].

## 2. Methods

### 2.1. Development of the Multivariable Index (MVI) for Scoring Gas Exchange Data

Based on previously reported data from our laboratory as well as others, we identified 6 variables that have been shown to track disease severity and/or prognosis in PAH and in the HF populations which can be obtained from rest and light, submaximal exercise [8–10, 13–18]. Many of these variables have published cut off values or ranges that are associated with higher risk [16, 19]. This includes (1) the ventilatory equivalents for carbon dioxide production (*V*_*E*_/*V*CO_2_) or breathing efficiency [19], (2) the oxygen uptake efficiency slope (OUES) [17], (3) oxygen saturation (SaO_2_) [20], (4) the resting PetCO_2_ [21], (5) the change in PetCO_2_ with exercise, and (6) a calculated gas exchange variable as an index for pulmonary capacitance (*P*_CAP_) which is the oxygen pulse multiplied by PetCO_2_ (O_2pulse_ × PetCO_2_) that tracks invasive measures of pulmonary capacitance [13] and a modifying variable based on the slope of change in the inflection of PetCO_2_ from rest to light exercise [12]. This final modifier has been suggested to reflect more severe exercise-induced changes in pulmonary vascular pressure and/or potential shunting through a PFO or intrapulmonary shunts due to high pressures [12]. There is some redundancy purposefully built into the MVI for variables most strongly associated with clinical measures, but yet retaining the ultimate goal of a single score that quantifies the severity of derangement in gas exchange rather than a formal surrogate to these other clinical markers. In fact, we would propose that in many cases that gas exchange data from light exercise may give a more important measure of integrated central hemodynamic function than the more commonly used “gold standards” for assessing and quantifying disease severity.

Table 1 describes the variable set used, the normal values [3, 4, 22, 23], and the delta value or the difference between the normal value and the risk cutoff point. In the lower table, the rows under “measured” are measured values of the variable in that column ranging in severity from normal to severe-very severe. The first column is individual variable index (IVI) score following severity, and the last column is cumulative IVI scores in a row. It is noted that some variables vary directly in severity from low to high (e.g., *V*_*E*_/*V*CO_2_ slope) and some variables vary in severity inversely from high to low (e.g., OUES). In this manner, if the measured = NV (normal value), the value of the IVI = 0. If the measured equals the risk cutoff point, the value of IVI = 1. IVIs that result in MVIs greater than 4.0 are scored as severe-very severe. Hence, MVI is cumulative IVI divided by 6. Normal subjects have MVI values less than 1.0, and it can be seen that the 6 variable MVI values closely resemble the NYHA classification system as shown in Table 2. 

### 2.2. Seven Variable Model with Weighting

Another feature of the MVI classification system is the ability to impart a greater weight to IVIs. It is proposed that this feature would allow for the evolution of disease specific MVIs. For this paper, the individual IVI for *P*_CAP_ was “double counted.” This metric was double weighted due to the ability of it to track pulmonary vascular capacitance, an important metric in gas exchange severity in both HF and PH. Therefore, the MVI was then obtained by dividing the cumulative IVI by 7, rather than 6 for the unweighted MVI. The effect of doing so can be observed in Table 3. 

### 2.3. Additional Modifiers

The MVI classification system also has the ability to apply modifiers. It has been demonstrated in the literature that an abrupt fall PetCO_2_ (steep slope) with exercise is itself a gauge of severity of PH [12]. We therefore increased the MVI score by values proportional to the magnitude and slope of change in PetCO_2_ during exercise (see Table 4). Adding the modifier for the PetCO_2_ patterns increased the severity score for individual subjects without altering the MVI scale range. In addition, adding the MVI_PH_ modifier to the MVI score consistently improved the correlations between the index and other clinical variables in both PAH and in the HF populations. 

## 3. Results

### 3.1. Testing the Model in Patient Groups

We examined the use of the final MVI score (CUM IVI/7 + additional modifier) in three populations from previously published studies (Table 5) [9, 24, 25]. This included patients with primarily PAH and classic systolic HF along with healthy subjects of similar age ranges. The PAH patients were recruited with known pulmonary hypertension through our PH clinic and performed a light submaximal 3 min step test after collecting 2 min of resting data, while the HF patients performed submaximal cycling ergometry (similar levels of perceived exertion). Control subjects performed a combination of the light step testing and submaximal cycle ergometry. Both patient groups had a range of disease severity levels and were typically on standard therapy. Breath by breath gas exchange data were collected for all populations using the Shape Medical Systems, Inc., simplified gas exchange system, and slopes (e.g., *V*_*E*_/*V*CO_2_) were determined by linear regression. Thirty second averages were used to calculate MVI variables. 

**Figure 1:**
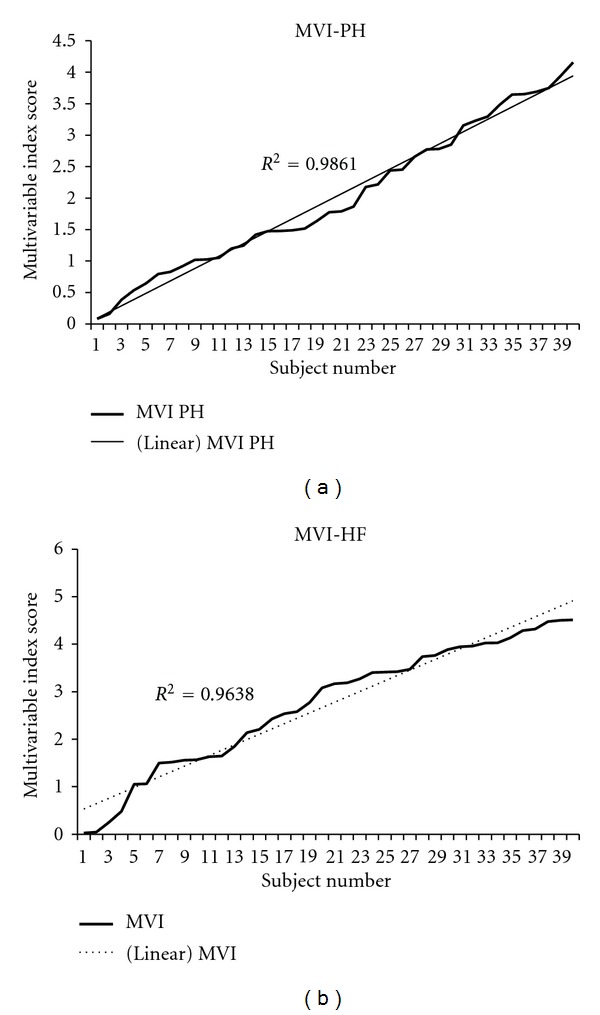
MVI score sorted and plotted for each subject for PAH and HF populations showing the score to be a continuous variable.

**Figure 2:**
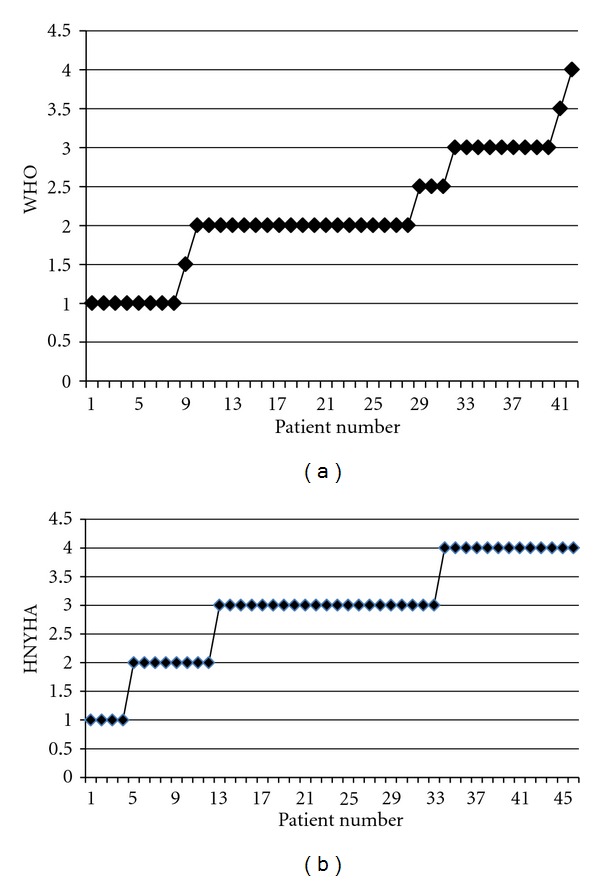
WHO classification for PH group and NYHA classification for HF group.

**Figure 3:**
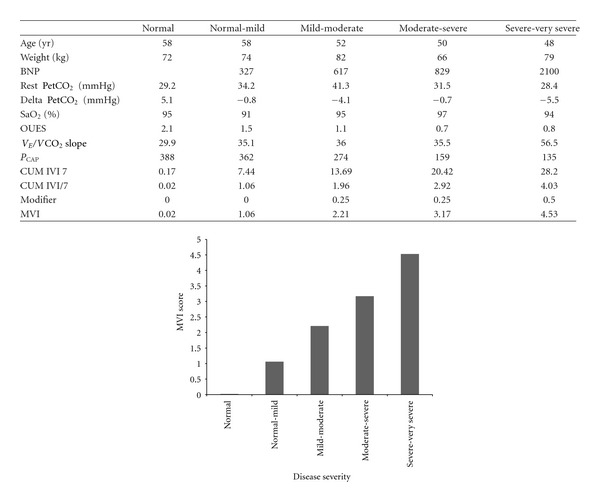
Final model with examples of *HF patients* according to gas exchange severity.

**Figure 4:**
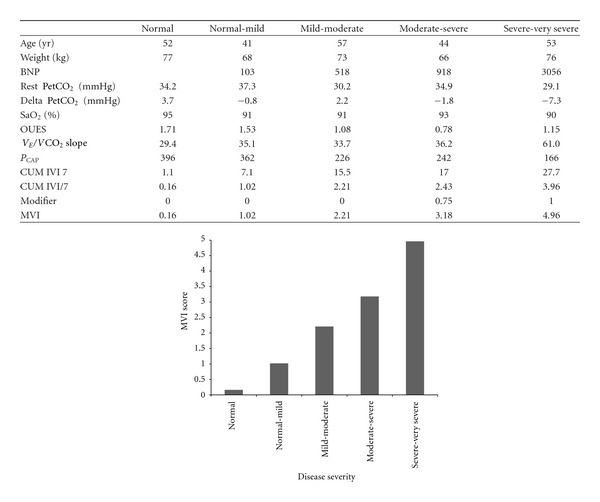
Final model with examples of *PAH patients* according to gas exchange severity.

**Figure 5:**
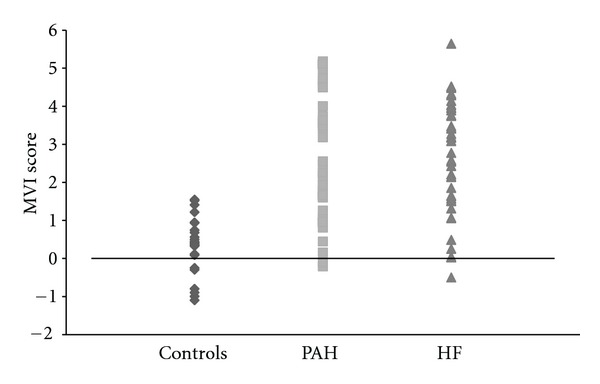
Range of MVI scores for patient groups and controls.

The ranges for MVI for each database are illustrated in Figure 1(a) (PAH) and Figure 1(b) (HF). When compared to the WHO or NYHA classification for the respective patient cohorts, (Figures 2(a) and 2(b)), it can be seen that the clinical classification results in “data aliasing” versus the MVI score which gives a continuous variable. Figures 3 and 4 give examples of individual PAH and HF patients over the range of scores obtained by the final MVI model. This also includes a healthy normal individual.  Figure 5 shows the ranges of MVI scores for the control, PAH, and HF populations. It should be noted that the patient populations presented have benefited from medical therapy, and thus overlap exists across populations. 

**Figure 6:**
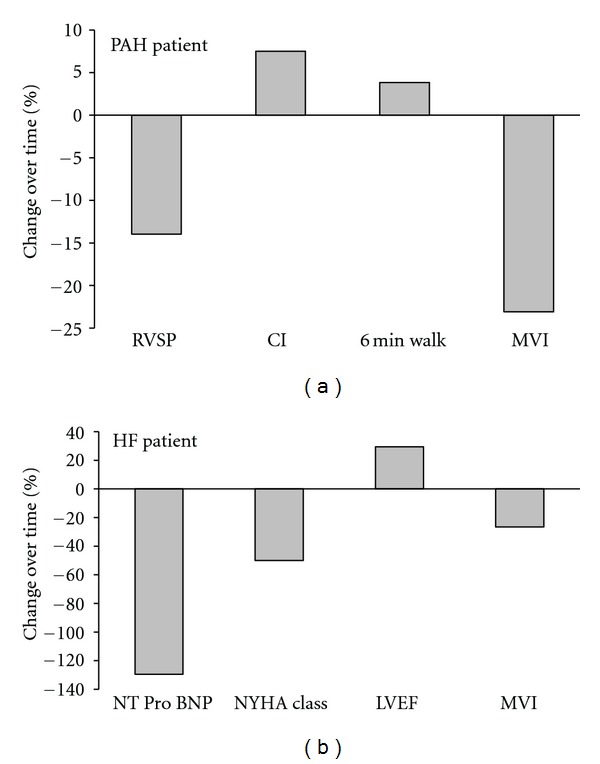
Tracking disease status over time, (a) PAH patient 3 mo. after treatment demonstrating modest improvements in clinical measures and the MVI score, (b) HF patients 3 mo. after CRT device implantation demonstrating similar directional changes in MVI score with clinical metrics.

**Figure 7:**
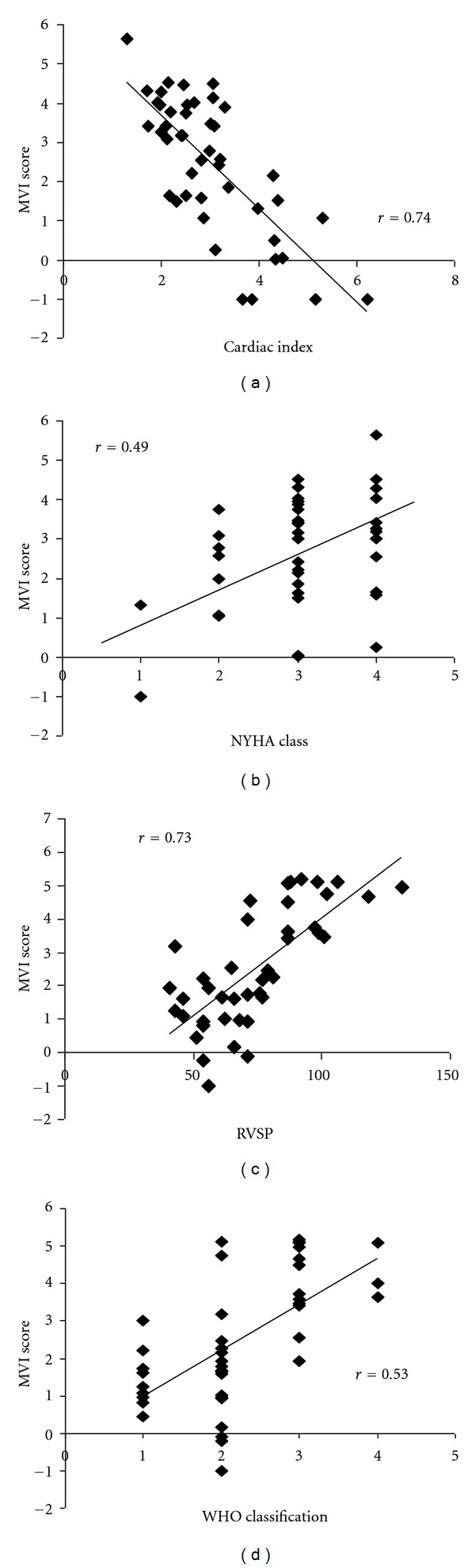
Relationships of MVI score with clinical parameters.

Figure 6 shows an example of a PH patient and an HF patient before and after intervention (medication titration in the PH patient and cardiac resynchronization therapy in the HF patient). Both patients demonstrated benefits in clinical measures (RVSP, CI, and 6 min walk in PH patient and NT Pro BNP, NYHA class, and LVEF in the HF patient), with improvements in the MVI score. We also examined the overall relationship between the MVI score to RVSP and WHO classification in PAH (Figures 7(c) and 7(d)) and CI and NYHA class in HF (Figures 7(a) and 7(b)). The MVI demonstrated a good relationship with clinical indices, (e.g., WHO class and RVSP in PAH *r* = 0.53 and 0.73, resp., and NYHA and CI in HF *r* = 0.49 and 0.74, resp.; *P* ≤ 0.01). The score was more highly correlated with the physiological measures versus the more subjective functional classifications. Individual correlations for the components of the MVI score with CI and RVSP are provided in Table 6. PetCO_2_, OUES, *V*_*E*_/*V*CO_2_ slope, and *P*_CAP_ all demonstrated significant relationships with CI in HF and RVSP in PAH patients with less significant relationships between these gas exchange measures and NYHA or WHO classification. Modest improvements over the majority of variables were observed using the MVI score. 

## 4. Discussion

### 4.1. Summary of Pertinent Findings

We propose a comprehensive multivariable index (MVI) scoring system to quantify gas exchange severity from light submaximal exercise data specific to populations with pulmonary vascular disease and demonstrate its utility in patients with PAH and systolic heart failure. The MVI allows a simple approach to integrating important gas exchange variables into a single conceptual score designed to track disease severity. The score is further weighted towards variables that reflect more severe hemodynamic derangement during exercise and is based on exercise loads that are commonly experienced by patients in daily activities. This score while designed to reflect gas exchange abnormalities and not necessarily other clinical tracking variables shows a modest association with clinically used classification schemes as well as catheter or echo-based measures.

### 4.2. Rationale and Goals for Designing a MVI Scoring System for Exercise Gas Exchange in Chronic Disease

While methods for capturing noninvasive gas exchange during exercise have evolved to simple breath by breath systems, little advancement has been made in simplifying the approach to interpretation and applying this to clinical populations. As a result, noninvasive measures of gas exchange during exercise remain underutilized in clinical practice [3, 4, 22]. A large number of variables are quantified during a typical test that include measures of breathing pattern, breath timing intervals, and gas exchange measures. Most established clinical exercise laboratories tend to focus on maximal testing and the classic assessment of peak oxygen consumption (*V*O_2peak_). However, there are a number of limitations in this type of assessment. This includes issues regarding patient anxiety with maximal testing-balance problems and uncertainties in their ability to push themselves to a true maximum. There is a need for more comprehensive monitoring equipment with the risks of maximal testing, often the need for multiple personnel for testing (increasing the cost), different use of protocols across centers as well as stopping criteria (making it hard to compare data), and the modest variability in the VO_2peak_ obtained. Over the last decade or more, it has become clear that a number of submaximal responses to exercise are as or more predictive for morbidity and mortality in the HF population, and many of these noninvasive submaximal measures are slopes or changes from rest and thus relatively insensitive to intensity of exercise, and in many cases being more reproducible [6]. Metrics that have been shown to be highly prognostic and sensitive to disease severity include the ventilatory efficiency, the oxygen uptake efficiency slope, the absolute or change in PetCO_2_, the change in O_2pulse_, (oxygen saturation, SaO_2_) [8–10, 13–18]. 

Ventilatory efficiency has been linked to high dead space ventilation, due mostly to a more rapid shallow breathing pattern, combined with a greater relative hyperventilation. It increases progressively with disease severity in both PAH and HF [8, 9, 26]. PetCO_2_ appears to track the rise in pulmonary vascular pressures with exercise, especially in PAH patients, likely not only due to both a pressure-induced increase in ventilation, but also due to increasing ventilation and perfusion inhomogeneities in the lungs and is typically inversely related with *V*_*E*_/*V*CO_2_ slope suggesting that in general they provide similar information [8, 9, 12]. Oxygen pulse (*V*O_2_/HR) is essentially the stroke volume multiplied times oxygen extraction, but appears to track stroke volume relatively well [13]. Using invasive or technical echocardiography-based measures, various techniques have been used to quantify a value representing pulmonary vascular capacitance (change in stroke volume relative to change in pulmonary pressures), which has been shown to be predictive of mortality in the PAH population [27, 28]. We previously compared a noninvasive estimate of pulmonary capacitance based on the equation (O_2pulse_, as an estimate of stroke volume) × (PetCO_2_, as an estimate of pulmonary vascular pressure) to catheter based measures obtained during exercise and found a strong relationship in the HF population [13]. The gas exchange derived *P*_CAP_ also demonstrated a relatively strong relationship with our clinical metrics in this study with only modest improvements using the complete MVI score. However, many gas exchange variables tend to change in concert, and in particular measures of PetCO_2_ and/or *V*_*E*_/*V*CO_2_ slope appear to be the variables that are most highly associated with clinical metrics and are counted or weighted heavily in the MVI scoring system, while at the same time allowing for other variables (e.g., SaO_2_) to contribute in a positive or negative way to the final score. In addition, such an approach to amalgamating variables tends to reduce noise. Thus the MVI score is weighted heavily towards factors which elevate dead space ventilation, inhibit a rise in stroke volume, cause a more rapid, and shallow breathing pattern and to a lesser extent cause oxygen desaturation with exercise (e.g., shunt, low VA/Qc regions, and diffusion limitation). We also amplify the negative score if the rate of change in PetCO_2_ with exercise is excessive.

### 4.3. Need for a Multivariable Gas Exchange Severity Score and Taking an Intuitive versus Statistical Approach

The MVI score demonstrates a modest improvement in the association with clinical measures over any single variable. However, while the score was purposefully weighted to track disease severity in the PAH and HF populations, the original intent was to create a gas exchange severity score and thus to some extent to be independent of other clinical measures. Thus, while one would expect the MVI score to generally track other clinical or physiological measures associated with disease severity, one would not necessarily expect a strong relationship with these clinical measures for a variety of reasons. For example, in some PAH patients, creating artificial shunts may reduce symptoms, but at the same time cause greater gas exchange abnormalities with exercise, making the gas exchange severity score worse. Therefore we chose to take an intuitive approach rather than a statistical approach to create the scoring system, as the score should be able to serve as an independent way to track disease and because there is no perfect gold standard for which to develop the statistical approach. In addition, other measures such as NYHA or WHO classification remain quite subjective. 

Other problems exist with the current “gold standards,” including a large variability in both echocardiogram and catheter-based measures, and both measures tend to have a number of limitations and often assumptions, particularly when cardiac hemodynamics are assessed during exercise. Thus our goal was to develop a comprehensive and adaptable gas exchange severity score based on the literature that is not dependent on maximal exercise values and provides an independent value for grading and tracking disease relative to other clinical measures.

### 4.4. Implications for the Future of Exercise Gas Exchange in Select Populations

With simplified techniques for quantifying gas exchange and the growing awareness that values obtained with light submaximal exercise are as prognostic as maximally obtained values in several populations, cardiopulmonary gas exchange could be easily adapted to many clinical areas as more of a “vital sign” rather than the more comprehensive and elaborate approach to testing that has classically been used, particularly in the HF and PH populations where ischemia detection is not a primary end point. Adding a gas exchange severity score to this simplified approach for screening and tracking patients further simplifies testing and reduces the need for specific expertise in cardio respirator physiology. We would propose that having a scoring system such as the MVI would allow a more comprehensive metric than “*V*O_2peak_” and a scaling system that is more similar to other scoring systems (e.g., NYHA or WHO classification) that are familiar to clinical experts. 

### 4.5. Limitations

We have created a gas exchange severity score that is weighted towards abnormalities in gas exchange found in HF and PAH. We have not specifically tested this in large populations with multiple comorbidities (e.g., COPD), and thus its utility in these patient groups would need to be determined. However, the MVI system is easily adaptable to other patient groups and changed or weighted towards additional variables that are more specific to a given population.

## 5. Conclusions

Measures of cardiopulmonary gas exchange with exercise have previously been underutilized due to their complexity and difficulty in interpretation. The MVI gas exchange severity score provides a simple means to rapidly assess disease risk and response to therapy in HF and PH patients and provides an overall assessment of integrative cardiac hemodynamics. The score reduces the complications of having to understand a large number of variables, eliminates the need for interpretation, accounts for variables with multiple directional changes, avoids noise that can be created by one value being abnormal versus the other values, and provides an easily identifiable numbering scheme for physicians to track.

## Figures and Tables

**Table 1:** Model showing individual variables (individual variable index, IVI) that make up the multivariable scoring system. Normal values from literature with delta representing a risk cutoff for each IVI. (MVI = CUM IVI/6).

	Rest PetCO_2_	ΔPetCO_2_	SaO_2_	OUES	*V*_*E*_/*V*CO_2_ slope	*P*_CAP_	
Normal value	40	3.6	94	1.6	26	400	
Delta	5	1.8	4	0.24	7	40	

Severity: IVI scores	Measured	Measured	Measured	Measured	Measured	Measured	CUM IVI

Normal: 0	40	3.6	94	1.6	26	360	0.00
Normal-mild: 1	35	1.8	90	1.36	33	320	6.00
Mild-moderate: 2	30	0	86	1.12	40	280	12.00
Moderate-severe: 3	25	−1.8	82	0.88	47	240	18.00
Severe-very severe: 4	20	−3.6	78	0.64	54	200	24.00

**Table 2:** Baseline multivariable index (MVI) scoring system.

CUM IVI	MVI = CUM IVI/6	Range	Severity	NYHA
0.00	0.00	<1	Normal	n/a
6.00	1.00	1 and <2	Normal-mild	I
12.00	2.00	2 and <3	Mild-moderate	II
18.00	3.00	3 and <4	Moderate-severe	III
24.00	4.00	≥4	Severe-very severe	IV

**Table 3:** MVI scoring system weighted for *P*_CAP_.

CUM IVI	MVI = CUM IVI/7	Range	Severity	NYHA
0.00	0.00	<1	Normal	n/a
7.00	1.00	1 and <2	Normal-mild	I
14.00	2.00	2 and <3	Mild-moderate	II
21.00	3.00	3 and <4	Moderate-severe	III
28.00	4.00	≥4	Severe-very severe	IV

**Table 4:** MVI scoring system weighted for the slope of change and magnitude of change in PetCO_2_ (indicative of exercise induced PH).

MVI_PH_	Modifier
≥0	0.00
<0 and >−5	0.50
≥−5 and >−10	0.75
≤−10	1.00

**Table 5:** Subject characteristics.

	Controls	PAH	Heart failure
Number (% female)	25 (80%)	40 (80%)	45/(13%)
Age (years)	51 ± 15	50 ± 13	54 ± 8
Height (cm)	167.8 ± 8.2	167.7 ± 7.0	174.9 ± 8
Weight (kg)	70.1 ± 12.7	75.8 ± 16.5	86.6 ± 16.3
HF etiology			
Ischemic/dilated (*n*)			23/22
NYHA Class (I/II/III/IV)			5/7/23/10
LVEF (%)	61 ± 7	64 ± 7.3	20 ± 6
NT Pro BNP/BNP		770 ± 1239	852 ± 2341
Cardiac index	3.0 ± 0.3	3.1 ± 0.7	1.9 ± 0.6
PAH etiology			
Idiopathic	—	25 (63%)	
Hereditary	—	4 (10%)	
Associated with diet drug use	—	2 (5%)	
Portopulmonary hypertension	—	1 (2%)	
Associated with connective tissue disease	—	8 (20%)	
Functional class (WHO) (I/II/III/IV)	—	7/20/11/2	
RV pressure (mmHg)	26 ± 4	76 ± 23	49 ± 18

**Table 6:** Relationship of individual gas exchange measures with CI and RVSP in HF and PAH, respectively.

	HF cardiac index	NYHA	PAH RVSP	WHO
Rest PetCO_2_	0.52	0.47	0.39	0.12
ΔPetCO_2_	0.42	0.23	0.62	0.55
SaO_2_	0.05	0.23	0.47	0.15
OUES	0.66	0.40	0.46	0.47
*V*_*E*_ /*V*CO_2_ slope	0.53	0.34	0.63	0.51
*P*_CAP_	0.69	0.40	0.58	0.41
MVI score	0.74	0.49	0.73	0.53
